# Syphilitic bicuspid aortic valve endocarditis diagnosed by metagenomic next-generation sequencing: a case report and literature review

**DOI:** 10.3389/fsurg.2026.1911873

**Published:** 2026-09-28

**Authors:** Zelei Ye, Yuxiang Liao, Zichen Gao, Shijia Chen, Qiaoqing Huang, Peifeng Jin

**Affiliations:** Department of Cardiac Surgery, The First Affiliated Hospital of Wenzhou Medical University, Wenzhou, Zhejiang, China

**Keywords:** cardiovascular syphilis, case report, culture-negative endocarditis, metagenomic sequencing, *Treponema pallidum*

## Abstract

**Background:**

Syphilitic endocarditis, a rare complication of tertiary syphilis, poses diagnostic challenges due to the unculturable nature of *Treponema pallidum* and non-specific clinical manifestations. This report highlights the role of quantitative metagenomic next-generation sequencing (Q-mNGS) in confirming the diagnosis.

**Case Presentation:**

A 52-year-old male patient with a bicuspid aortic valve presented with a cough and chest tightness. Serology confirmed syphilis, but blood cultures were negative. Transesophageal echocardiography revealed severe aortic regurgitation with vegetations. Q-mNGS of resected valve tissue detected *T. pallidum* despite negative Warthin–Starry staining. The patient underwent valve replacement and received penicillin G, with no recurrence at the 2-year follow-up.

**Conclusions:**

Q-mNGS shows potentially higher sensitivity for diagnosing syphilitic endocarditis compared to traditional methods. Serological screening combined with molecular techniques is critical for culture-negative endocarditis in high-risk populations.

## Background

Syphilis, caused by *Treponema pallidum*, remains a global health concern despite penicillin efficacy (1). Cardiovascular manifestations (e.g., aortitis and aneurysms) occur in 10% of untreated cases (2), but endocarditis is exceptionally rare, with <5 cases reported in the post-antibiotic era (3, 4). Diagnostic hurdles include the following: (1) *T. pallidum* cannot be cultured, and histological staining sensitivity is ≤50% (5, 6); (2) symptoms overlap with bacterial endocarditis, often delaying diagnosis (7, 8). Quantitative metagenomic next-generation sequencing (Q-mNGS) offers a paradigm shift by detecting microbial DNA directly from tissue, yet its application in syphilitic endocarditis is undocumented prior to this case.

## Case report

A 52-year-old man with no syphilis history presented with a 1-month cough and 2-week chest tightness. The patient had no fever or rash during the previous month. A diastolic murmur was heard in the aortic area. Laboratory results indicated an elevated brain natriuretic peptide level (BNP, 715 pg/mL) and normal anti-streptolysin O and rheumatoid factor levels. Transesophageal echocardiography showed a bicuspid aortic valve (BAV) with vegetations and severe regurgitation, as well as severe mitral valve and moderate tricuspid valve regurgitation (Figures 1A–C). Three sets of blood cultures yielded negative results, but a serum chemiluminescence immunoassay was positive and the toluidine red unheated serum test (TRUST) titer was 1:4. Endocarditis was suspected based on characteristic imaging and laboratory findings.

**Figure 1:**
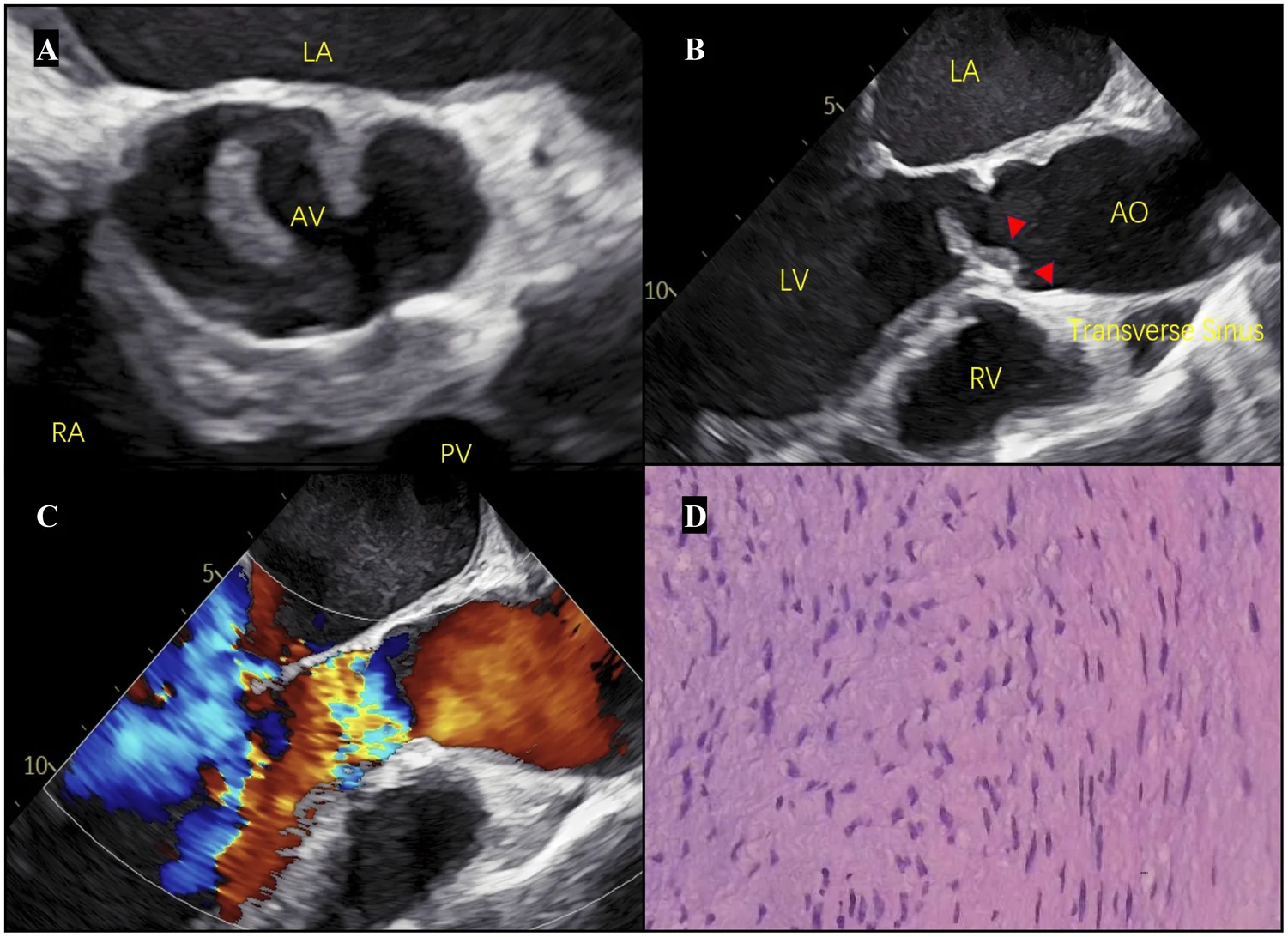
Transesophageal echocardiography shows a bicuspid aortic valve **(A)**, a vegetation on the aortic valve **(B)**, and severe aortic and mitral valve regurgitation **(C)**. Histopathology shows fibrosis and mucoid degeneration in the aortic valve **(D)**. AV, aortic valve; RA, right atrium; LA, left atrium; PV, pulmonary valve; LV, left ventricle; RV, right ventricle; AO, aorta.

Owing to concern about the severe disease burden, the patient underwent mechanical aortic valve replacement, mitral valve replacement, and tricuspid valve repair. The intraoperative inspection revealed aortic dilatation (4.0 cm) with wall thickening and aortic valve perforation (0.4 cm) with vegetations. Resected aortic valve tissues were sent to pathology and microbiology for further analysis.

Histopathology showed fibrosis, mucoid degeneration, and calcification in the aortic valve (Figure 1D). The Warthin–Starry staining was negative. Illumina-based Q-mNGS of aortic tissue identified 394 reads uniquely mapped to the *T. pallidum* reference genome (NC_021508.1, NCBI RefSeq), representing 25.96% microbial relative abundance.

The negative blood culture results, the positive serological tests, and the positive Q-mNGS result indicated that *T. pallidum* was the pathogen responsible for the infection. A final diagnosis of syphilitic endocarditis was made. Thus, we administered 4 million units of intravenous penicillin G every 4 h for 14 days. Finally, the cough and chest tightness were significantly relieved. There was no recurrence at the 2-year follow-up visit.

## Discussion

Syphilitic endocarditis remains a diagnostic enigma in the era of modern medicine. Our case underscores the following three critical issues: (1) the limitations of conventional diagnostic approaches; (2) the transformative role of Q-mNGS in pathogen detection; and (3) the therapeutic implications of this rare yet devastating complication.

Microbiology and imaging are the preoperative clinical criteria for endocarditis (8). For blood culture-negative endocarditis, pathology is the most reliable evidence for a definitive diagnosis. However, the sensitivity of postoperative valve culture and staining is 17% and 52%, respectively (9). In a review of the literature, the use of the modified Duke's criterion was found to diagnose only 32% of culture-negative endocarditis cases (10). Q-mNGS is a high-throughput technique encompassing genome-wide areas, possessing high sensitivity (97.6%), high efficiency, and high negative predictive value (9, 11, 12). Q-mNGS plays a pivotal role in pathogen identification, ultimately improving clinical outcomes. The application of Q-mNGS for diagnosing syphilitic endocarditis represents an unprecedented approach.

Compared with other cardiovascular syphilis complications (e.g., aortitis and aneurysms), syphilitic endocarditis is underreported. Only a few cases of syphilitic endocarditis have been documented. The reasons for this may include the following: (1) the use of antibiotics has dramatically reduced the prevalence of syphilis (1); (2) cardiovascular syphilis occurs in only 10% of untreated syphilis cases (2); (3) valvular surface lesions are a prerequisite for infective endocarditis (7); (4) incomplete diagnostic criteria and inadequate diagnostic techniques hinder the diagnosis of culture-negative endocarditis (8–10); and (5) case scarcity has resulted in inadequate attention and awareness. Abnormal hemodynamics in BAV promote endothelial injury and microbial adhesion, leading to a 12-fold increased risk of endocarditis and increased incidence of complications compared with tricuspid aortic valves (13). The complications of untreated syphilis with abnormal valves still warrant clinical vigilance.

Penicillin remains the treatment of choice for syphilis, with no reported cases of *T. pallidum* resistance (1). For treatment of cardiovascular syphilis, the US Centers for Disease Control and Prevention and European guidelines recommend three doses of intramuscular Benzathine penicillin G 2.4 million units 1 week apart. For neurosyphilis, 3–4 million units of penicillin G is administered intravenously every 4 h or in a continuous infusion for 10–14 days (14, 15). After consultation with an infectious disease specialist, cardiovascular syphilis can be treated with a neurosyphilis regimen (15, 16). Therefore, we ultimately administered a neurosyphilis treatment regimen.

Surgical intervention at the optimal time improves outcomes in certain endocarditis cases. The American Association for Thoracic Surgery and European Society of Cardiology guidelines recommend surgery for endocarditis with heart failure and uncontrolled infection and for prevention of septic embolization. Endocarditis with valve dysfunction resulting in heart failure symptoms provides a strong surgical indication (17, 18). In this case, severe aortic and mitral valve regurgitation with heart failure symptoms necessitated surgical intervention. As expected, surgery significantly alleviated the patient’s symptoms and enhanced prognosis.

A limitation of this report is the failure to detect *T. pallidum* by traditional staining due to its insufficient sensitivity (5, 6, 9). Additionally, etiological testing of the aorta and mitral valve was not performed, as the aorta was not replaced (diameter <4.5 cm) and no vegetations were seen on the mitral valve, so endocarditis was not suspected there.

## Conclusions

This case illustrates that syphilitic endocarditis is insidious and dangerous. Therefore, simple and inexpensive serological testing is warranted for high-risk patients with negative blood cultures. To overcome the shortcomings of traditional laboratory tests, the more sensitive and faster Q-mNGS can be used for definitive diagnosis. In conclusion, the combination of serological screening and molecular testing techniques may mitigate the diagnostic challenges and improve patient prognosis.

## Data Availability

The original contributions presented in the study are included in the article/Supplementary Material, further inquiries can be directed to the corresponding author.
