# Supplementary material for: Syphilitic bicuspid aortic valve endocarditis diagnosed by metagenomic next-generation sequencing: a case report and literature review
Source: Front Surg. 2026 Sep 28;13:1911873. doi: 10.3389/fsurg.2026.1911873 (PMC13655015; doi:10.3389/fsurg.2026.1911873)
Supplement: Supplementary file 1 [file Supplementaryfile1.docx]

**Experimental method:**

Collected samples were subjected to metagenomic next-generation sequencing (mNGS). Briefly, nucleic acids were extracted from samples using an automated nucleic acid extraction system. The extracted nucleic acids were subsequently processed using the NGSmaster™ automated workstation for library preparation, including reverse transcription (for RNA samples only), nucleic acid fragmentation, end repair, 3′ adenylation (addition of a single adenosine residue to the 3′ end), adapter ligation, and purification to generate sequencing libraries.

The prepared libraries were quantified using a quantitative fluorescence PCR instrument and subsequently subjected to shotgun sequencing on an Illumina NextSeq™ high-throughput sequencing platform.

Bioinformatic analysis was performed on the generated sequencing reads. Human-derived sequencing reads were removed by alignment against the human reference genome (GRCh38.p13). The remaining non-human sequences were aligned against microbial reference databases, including the NCBI GenBank database and an internally curated microbial genome database, for microbial identification and estimation of relative abundance.

Each mNGS run included both negative and positive controls. The negative control consisted of a mixture of plasma cell-free nucleic acids and fragmented human genomic DNA, while the positive control contained a mixture of inactivated bacterial, fungal, and pseudoviral particles.

**mNGS quality control and reporting criteria**

Sequencing data were considered qualified when the following quality control criteria were met: Q20 > 85% and Q30 > 80%.

To distinguish true-positive microbial signals from potential background contamination, negative controls (NCs) were included in each sequencing batch. A microbial species was considered positively detected when it was absent in the corresponding negative control or when the relative abundance ratio between the sample and negative control met the predefined threshold:

RPM (sample) / RPM (NC) ≥ 5

where RPM represents reads per million sequencing reads.

**Sample extraction, purification and enrichment**
Samples were loaded into a nucleic acid extraction kit for automated nucleic acid extraction. Extracted nucleic acids were further processed using a nucleic acid extraction and purification kit for enrichment, purification, and recovery of high-quality nucleic acid products.

Principle of the extraction procedure: In the presence of lysis buffer and proteinase, cells are lysed and proteins are denatured, resulting in the release of nucleic acids. Under high concentrations of chaotropic agents, magnetic beads specifically bind nucleic acids through hydrogen bonding and electrostatic interactions. Proteins and other nonspecifically adsorbed impurities are removed through washing steps. Finally, purified nucleic acids are eluted from the magnetic beads using a low-salt buffer or elution solution.

Principle of the purification procedure: Human-derived cells were lysed in the presence of lysis buffer and proteinase K, resulting in protein denaturation and release of nucleic acids. The released nucleic acids were specifically captured by superparamagnetic beads. Subsequent washing steps removed contaminants, including proteins, polysaccharides, and other impurities. Purified nucleic acids were subsequently released from the magnetic beads using an elution buffer, yielding nucleic acid products with high purity and concentration.
